# Harnessing multiplex PCR assay targeting specific mitochondrial DNA elements for simultaneous identification of antelope species in *Cornu Saigae Tataricae*

**DOI:** 10.1080/23802359.2019.1666667

**Published:** 2019-09-25

**Authors:** Yufei Chen, Yaya Yang, Yinhuan Qian, Roselyn Tehzee Gblinwon, Zhaoqun Jiao, Liqun Chen, Ling Lin, Yang Zheng, Huan Yang, Yuping Shen

**Affiliations:** a School of Pharmacy, Jiangsu University, Zhenjiang, Jiangsu, China;; b Department of Pharmacy, Zhenjiang Hospital of Traditional Chinese Medicine, Zhenjiang, Jiangsu, China

**Keywords:** Mitochondrial DNA, species-specific primer, multiplex PCR assay, antelope horn, animal-derived Chinese medicine, species identification

## Abstract

A multiplex PCR assay was developed to simultaneously differentiate four antelope species and identify adulteration in *Cornu Saigae Tataricae*. Four novel primer sets were designed with high inter-species specificity and intra-species stability. Limit of detection was estimated to be 10 ng of genomes. When a mixture of antelope hornand fake species was assayed, it exhibited powerful differentiation capability. 5 out of 12 batches of commercialproducts were identified to be counterfeited or adulterated with *Ovis aries* Linnaeus and/or *Capra hircus* Linnaeus. It is readily applicable in routine analysis for identification of sham or adulterants of *Cornu Saigae Tataricae*.

## 1. Introduction

*Cornu Saigae Tataricae*, also known as antelope horn, is a famous and precious Chinese Medicine made of *Saiga tatarica* Linnaeus (STL) horn. It has been widely used for thousand years due to its significant curative effect on the endogenous liver wind, epileptic seizures, and high fever (Chinese Pharmacopoeia Committee 2015). In addition, modern pharmacological studies have shown that it has functions of hypnosis, analgesis, anti-inflammation, and immunoenhancement (Costa-Neto 2005).

Nowadays, this animal origin medicine is limited, and counterfeit or adulteration is a serious problem in market. For example, the horn of *Ovis aries* Linnaeus (OAL), *Capra hircus* Linnaeus (CHL), and *Procapra gutturosa* Pallas (PGP) were sold as genuine products. To date, protein and peptide analyses have been employed for species detection of natural products (Yang et al. 2015, 2017; Shen et al. 2019), but complicated procedures and expensive equipment are usually needed, and reliable identification of a mixture can’t be readily achieved due to similar chemical properties. In recent decades, PCR is well recognized, to be more specific, sensitive, and even applicable to processed products, and has been widely used in such applications (Chen et al. 2011; Zhang et al. 2016). And real-time PCR (Asing et al. 2016) and species-specific PCR (Zhu et al. 2015; Duan et al. 2017; Yang et al. 2018; Yang, Zhou, et al. 2019; Zheng et al. 2019) were also developed for their authentication. Compared with them, multiplex PCR has proved to be the most practical technique offering a convenient, specific, and simultaneous detection of more than one species (Prusakova et al. 2018; Chen et al. 2019; Yang, Zheng, et al. 2019).

**Figure 1.**
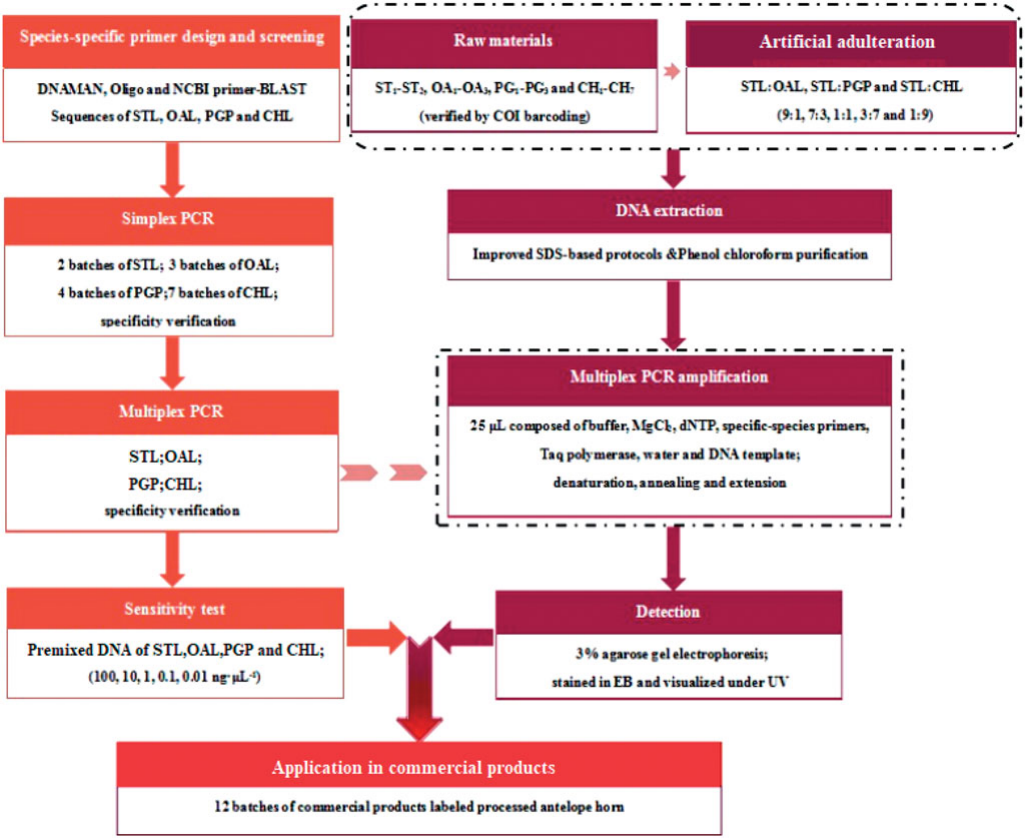
Flow chart for the establishment of the multiplex PCR assay.

Many kinds of research have demonstrated that conservative *mt*DNA of maternal inheritance without complicated intron, pseudogene or repetitive sequence was easier to analyze (Ludt et al. 2004). Consequently, specific primers were designed on the basis of the differences of species *mt*DNA and have been the first choice for the identification of target species in a PCR assay (Ghovvati et al. 2009; Zha et al. 2011; Li et al. 2017; Ma et al. 2019; Yuan et al. 2019). In the present study, four specific primer sets were designed according to both intraspecific homology and interspecific variation in mitochondrial complete genome of STL, OAL, CHL, and PGP, which enabled the formation of amplified products specific for four target antelope species in the multiplex PCR assay and, herewith, the use of gel electrophoresis for their sufficient separation. Furthermore, a novel non-sequencing and specific multiplex PCR approach were established for simultaneous identification of four antelope species, followed by validation for specificity, sensitivity, and applicability to adulteration, which was eventually used for reliable and convenient authentication of commercially processed antelope horn products (Figure 1).

## 2. Materials and methods

### 2.1. Samples

Sixteen batches of the raw material and 12 batches of commercial products was collected and relevant information of them is listed in Table S2. These samples were smashed into their fine powder in liquid N_2_, and then subject to genomic DNA extraction. The purity and concentration of the extracted DNA were quantified and were then diluted to 100 ng μL^−1^ as template for further PCR assays.

### 2.2. Methods

Specific target genetic elements for the species identification of antelope horn products were mined by the comparative genomics approach. The designed primer pairs were then screened according to the principle including possible consistence of base composition and annealing temperature of each primer set, high intraspecies-universality and interspecies-specificity, the reliable score of primers, and ideal length of the amplified product.

In a preliminary phase of this research, primers specificity was evaluated on the DNA extracts of the species panel listed in Table S2. And the resulting products after the PCR amplification were subject to DNA sequencing to verify the specificity of those four primer sets and confirm species identity of amplicons.

On this basis, a one-step multiplex PCR assay for simultaneous species detection was developed using four primer sets previously screened by the simplex PCR. To achieve the best detection result, the elementary reaction system was necessarily optimized. After extraction and purification of the genomic DNA of each antelope species and analyzing the concentration of DNA extract, the developed multiplex PCR assay was validated for sensitivity against four primer sets, which were further amplified to determine the minimal concentration that can be detected.

Finally, analysis of artificial adulteration antelope horn and application of multiplex PCR assay to commercially processed antelope horn products were conducted to verify the feasibility of the established method.

More description of materials and methods can be found in ‘Supplemental Online Material’.

## 3. Results and discussion

### 3.1. Screening of primer sets specific for antelope species

Seventeen of the candidate primer sets were designed by DNAMAN and Oligo software (Table S1). To evaluate their specificity, certified raw materials of STL, OAL, PGP, and CHL (Table S2) were tested. The simplex PCR result showed that four primer sets (Table 1) appeared to be much specific against their corresponding antelope species and other thirteen primer sets did not show the good specificity (Figure S1). All of them were then selected for PCR amplification in the subsequent experiments and synthesized by Shanghai Sangon Co., Ltd.

**Table 1.** Primer sets selected for PCR assay in this study.

Species	Code		Sequence (5’-3’)	Target gene	Length (bp)
STL	PST	F	ACTTCTAGCATCTTCCATAGTTGAG	COX1	266
		R	GGGAAGTGAAAGGAGTAGGAGG		
OAL	POA	F	TGGCATTCACAGTATCCCT	ND4L	245
		R	TTGTACATAGTCGGTGCCAT		
PGP	PPG	F	TTATCTGGCATACCACGAC	COX1	159
		R	TACAGTTGAGACTTCCCGTT		
CHL	PCH	F	GCCGAACTAGGTCAACCC	COX1	130
		R	GTCAGTTGCCAAACCCTC		

Furthermore, simplex PCRs were performed on the DNA extracted from all 16 batches of certified raw materials to verify the specificity. As shown in Figure S2, PST, POA, PPG, or PCH has individually amplified specific fragments of 266, 245, 159, or 130 bp for the species STL, OAL, PGP, or CHL, respectively. The results of various batches of these four antelope species adequately demonstrated that each primer set can produce its own species-specific bands. Meanwhile, the amplicon sequences can be aligned with their corresponding species, which suggested that the specific primers used in this study were reliable and feasible (Figure S3).

### 3.2. Optimized multiplex PCR assay

From Figure S4(a), it could be seen that when the dNTP addition was 0.4 mM, the four specific bands for STL, OAL, PGP, and CHL became clear, suggesting that the dNTP concentration was proper. On the other hand, the effect of Taq polymerase additions on the PCR product was investigated. The result was shown in Figure S4(b), and it can be found that four specific bands for all antelope species were bright and clear when Taq enzyme addition was increased from 0.625 unit to 1.025 unit or 1.225 unit. Accordingly, the addition of 1.025 unit of Taq polymerase was optimal.

Under the optimized PCR reaction conditions, the multiplex PCR assay was eventually carried out on the premixed DNA templates of ST1, OA1, PG1, and CH1. As shown in Figure S5, four bright and clear bands, located at positions of 266, 245, 159, and 130 bp, have been successfully achieved. The primer sets still maintained the analogous specificity as the simplex PCR assay, and the electrophoresis pattern obviously presented the absence of cross-reactions. The results implied that the primer sets PST, POA, PPG, and PCH were of high specificity for STL, OAL, PGP, and CHL respectively, and they could clearly differentiate these four antelope species by the multiplex PCR amplification.

### 3.3. Sensitivity evaluation of the multiplex PCR assay

As shown in Figure S6, when the final concentration of the premixed DNA template was decreased from 100 to 10 ng μL^−1^, the four specific bands have been clearly achieved but difficult to observe with further 10-fold decrement of the template concentration to 1, 0.1, and 0.01 ng μL^−1^. Accordingly, the detection limit was determined as 10 ng μL^−1^.

### 3.4. Analysis of artificial adulteration antelope horn

As shown in Figure S7, antelope origins in fifteen artificial adulteration antelope horns of different proportions were well detected by their corresponding specific primers via the newly developed multiplex PCR.

It can be seen that the band intensity of the two mixtures (STL:PGP and STL:CHL) presented an increasing or decreasing trend with component ratios varied from 9:1 to 1:9. However, the bands of similar brightness were obtained by the primer set POA when the first mixtures (STL:OAL) was subject to the multiplex PCR amplification, meanwhile, the intensity of the specific band amplified by the other primer set PST was gradually reduced to marginal existence. In general, the amplified bands were relatively bright and clear at 10% adulteration level, which also indicates that the developed multiplex PCR assay with the primer sets PST, PPG, POA, and PCH could be applicable to the identification of adulterated antelope horn products.

### 3.5. Application of multiplex PCR assay to commercially processed antelope horn products

**Figure 2.**
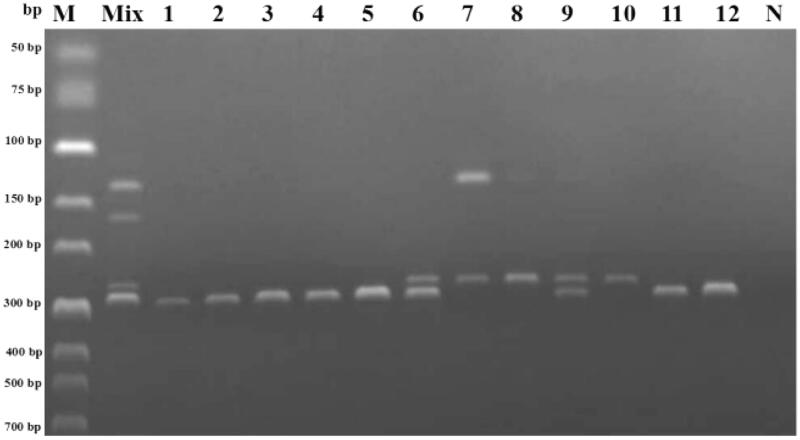
Analysis of twelve batches of commercial processed antelope horn products by the multiplex PCR. M: DNA marker; Mix: premixed DNA of STL, OAL, PGP, and CHL; N: negative control.

Twelve batches of antelope horn (Table S3) were subjected to DNA extraction and purification. The optimized PCR reaction conditions were then applied to the sample extracts for identification of antelope species and verification of labeling compliance. The results could be seen in Figure 2 and were summarized in Table S4.

According to the results, all DNA templates extracted from commercially processed products were successfully amplified by the multiplex PCR assay. It was found that seven (Nos. 1–5; Nos. 11 and 12) of commercial products were amplified solely by STL. Thus, these results were consistent with the labeling information provided by the manufacturers. However, one of the rest five batches (No. 10) were amplified by POA only, and positive results were also detected in another two batches (Nos. 7 and 8) using POA and PCH. Thus, these three batches were identified to be counterfeited by OAL and CHL. Additionally, specific bands of PST were shown after the assay for batches 6 and 9, however, a bright and clear band of POA was observed too, and even a faint band of PCH in the sample No. 9. Therefore, these two batches were considered as adulterated products with OAL or OAL/CHL. None of the 12 batches have been amplified by PPG.

The results above indicated that all of the counterfeit or adulterated products were made from antelope species OAL and CHL horn but not PGP. The most frequent adulteration revealed was substitution by OAL, which is, apparently, a vivid illustration of economically motivated adulteration. As the contamination during manufacturing or sale probably could not have taken place, deliberate adulteration is the only explanation for trace amounts of amplified PCR fragments of CHL detected in sample nos. 8 and 9.

## 4. Conclusions

In this study, four pairs of novel primer set (PST, POA, PCH, and PPG) was designed. Furthermore, a multiplex PCR assay was developed to rapidly and reliably identify the antelope species in commercial processed *Cornu Saigae Tataricae* products. The newly established method proved to be sensitive and reliable in simultaneous identification of antelope species, in addition to a low adulteration level at 10% when the artificially adulterated products were tested. It was also found that the DNA fragments from OAL and CHL have been frequently detected in the counterfeited or adulterated products but not PGP, which indicated the substitution of horn species by OAL and CHL was rather prevalent in market and probably due to intentional admixture for economic reasons.

## Supplementary Material

Supplemental Material
