# Supplementary material for: Harnessing multiplex PCR assay targeting specific mitochondrial DNA elements for simultaneous identification of antelope species in Cornu Saigae Tataricae
Source: Mitochondrial DNA B Resour. 2019 Sep 25;4(2):3222–6. doi: 10.1080/23802359.2019.1666667 (PMC7707849; doi:10.1080/23802359.2019.1666667)
Supplement: Supplemental Material [file TMDN_A_1666667_SM3225.zip › Huan Yang et al supplemental content.doc]

SUPPLEMENTARY MATERIAL

Harnessing Multiplex PCR Assay Targeting Specific Mitochondrial DNA Elements for Simultaneous Identification of Antelope Species in *Cornu Saigae* Tataricae

Yufei Chen^a+^, Yaya Yang^a+^, Yinhuan Qian^b^, Roselyn Tehzee Gblinwon^a^, Zhaoqun Jiao^a^, Liqun Chen^a^, Ling Lin^a^, Yang Zheng^a^, Huan Yang^a,*^, and Yuping Shen^a,*^

^a^ School of Pharmacy, Jiangsu University, Zhenjiang 212013, China

^b^ Department of Pharmacy, Zhenjiang Hospital of Traditional Chinese Medicine, Zhenjiang 212003, China

*Corresponding authors: School of Pharmacy, Jiangsu University, Zhenjiang 212013, Jiangsu Province, China. Tel.: +86 (511) 85038170; Fax: +86 (511) 88795939.

E-mail addresses: syp131@ujs.edu.cn (Yuping Shen); yanghuan1980@ujs.edu.cn (Huan Yang).

**Table S1. Seventeen candidate primer sets**

| **Species** | **Code** | | **Sequence (5’-3’)** | **Target gene** | **Length (bp)** |
| --- | --- | --- | --- | --- | --- |
| STL | PST-1 | F | ACTTCTAGCATCTTCCATAGTTGAG | COX1 | 266 bp |
|  |  | R | GGGAAGTGAAAGGAGTAGGAGG |  |  |
| STL | PST-2 | F | GCTAAATTACAGGTGAAAACC | COX2 | 204 bp |
|  |  | R | CGCGTCTATCGTGCTAGTATGT |  |  |
| STL | PST-3 | F | ACTTCCCGTACTAGCTG | COX1 | 169 bp |
|  |  | R | AATTATTCCAAATCCGGGTA |  |  |
| OAL | POA-1 | F | TAACCCACATTTGCCGAGA | CYTB | 75 bp |
|  |  | R | AAAATATTGATGCCCCGTT |  |  |
| OAL | POA-2 | F | AGGAACCGCCTTAAGCCTA | COX1 | 162 bp |
|  |  | R | ACTAGTCAGTTGCCGAAT |  |  |
| OAL | POA-3 | F | TATCGGCACCCTTTACCTTC | COX1 | 141 bp |
|  |  | R | TGTGCGGTTACAATTACGTT |  |  |
| OAL | POA-4 | F | AGGAACCGCCTTAAGCCTA | COX1 | 96 bp |
|  |  | R | TGTGCGGTTACAATTACGTT |  |  |
| OAL | POA-5 | F | TGGCATTCACAGTATCCCT | ND4L | 245 bp |
|  |  | R | TTGTACATAGTCGGTGCCAT |  |  |
| OAL | POA-6 | F | CCTATTCATTACCATCGCACT | COX3 | 193 bp |
|  |  | R | ATGAAATTTTAATTGGCGGAA |  |  |
| PGP | PPG-1 | F | CGGCATACTACGAATCACAC | ND4 | 230 bp |
|  |  | R | CAATTATTAAGGCGGTAGCTC |  |  |
| PGP | PPG-2 | F | TAGAAGGAAATCGCAACCA | COX3 | 68 bp |
|  |  | R | AGTAAAATAAACACCGAGT |  |  |
| PGP | PPG-3 | F | TTATCTGGCATACCACGAC | COX1 | 159 bp |
|  |  | R | TACAGTTGAGACTTCCCGTT |  |  |
| CHL | PCH-1 | F | ACAATACTCATCCTCGTCACC | ND5 | 106 bp |
|  |  | R | TTCGTCCATATCACCATCCG |  |  |
| CHL | PCH-2 | F | CCTTAATCCAATCACCGACT | ND4 | 193 bp |
|  |  | R | AGTGGCTCCTATGTAGCTTC |  |  |
| CHL | PCH-3 | F | GCCGAACTAGGTCAACCC | COX1 | 130 bp |
|  |  | R | GTCAGTTGCCAAACCCTC |  |  |
| CHL | PCH-4 | F | CCTTATGTCGACTCGTCCAG | COX2 | 94 bp |
|  |  | R | AACTAGCTCGAGAACGATT |  |  |
| CHL | PCH-5 | F | ACGGTCTTCATGTTATCATCGG | COX3 | 112 bp |
|  |  | R | TATCAGGCAGCAGCTTCGAAA |  |  |

**Table S2. Raw materials of horn species used in the study.**

| **Internal Code** | **Species** | **Sources** | **Collection Time** |
| --- | --- | --- | --- |
| ST_1_ | STL | NIFDC, PRC | April, 2017 |
| ST_2_ | STL | NIFDC, PRC | June, 2017 |
| OA_1_ | OAL | City of Bengbu, Anhui Province, PRC | July, 2018 |
| OA_2_ | OAL | City of Bengbu, Anhui Province, PRC | July, 2018 |
| OA_3_ | OAL | City of Linyi, Shandong Province, PRC | July, 2018 |
| PG_1_ | PGP | City of Tieling, Liaoning Province, PRC | May, 2018 |
| PG_2_ | PGP | City of Nanyang, Henan Province, PRC | June, 2018 |
| PG_3_ | PGP | City of Nanyang, Henan Province, PRC | July, 2018 |
| PG_4_ | PGP | City of Zhengzhou, Henan Province, PRC | July, 2018 |
| CH_1_ | CHL | City of Baoding, Hebei Province, PRC | April, 2018 |
| CH_2_ | CHL | City of Dalian, Liaoning Province, PRC | April, 2018 |
| CH_3_ | CHL | City of Bengbu, Anhui Province, PRC | May, 2018 |
| CH_4_ | CHL | City of Bengbu, Anhui Province, PRC | June, 2018 |
| CH_5_ | CHL | City of Bengbu, Anhui Province, PRC | July, 2018 |
| CH_6_ | CHL | City of Bengbu, Anhui Province, PRC | July, 2018 |
| CH_7_ | CHL | City of Zhenjiang Province, Jiangsu, PRC | August, 2018 |

**Figure S1.** Screening of primer sets specific for four antelope species

**Figure S2.** Specificity test of four specific primer sets by the simplex PCR. M: DNA marker; ST_1_~ST_2_: *Saiga tatarica* Linnaeus; OA_1_~OA_3_: *Ovis aries* Linnaeus; PG_1_~PG_4_: *Procapra gutturosa* Pallas; CH_1_~CH_7_: *Capra hircus* Linnaeus; N: Negative control.

**Figure S3.** Amplicons of the specific primer sets sequenced and aligned.

**Figure S4.** Optimization of dNTP addition (a), A~D: 0.2 mM, 0.4 mM, 0.6 mM, 0.8 mM; Taq polymerase amount (b), E~H: 0.625 U, 0.825 U, 1.205 U, 1.225 U

**Figure S5.** Specificity test of the multiplex PCR assay. M: DNA marker; Mix: premixed DNA of *Saiga tatarica* Linnaeus, *Ovis aries* Linnaeus, *Procapra gutturosa* Pallas and *Capra hircus* Linnaeus; N: Negative control.

**Figure S6.** Sensitivity test of the multiplex PCR. The concentration of premixed DNA from lane S1 to lane S5 was 100 ng·μL^-1^, 10 ng·μL^-1^, 1 ng·μL^-1^, 0.1 ng·μL^-1^, 0.01 ng·μL^-1^; M: DNA marker; N: Negative control.

**Figure S7.** Analysis of artificial adulteration antelope horn by the multiplex PCR. M: DNA marker; Mix: premixed DNA of STL, OAL, PGP and CHL; A~E: five ratios (9:1, 7:3, 1:1, 3:7, 1:9); N: Negative control.

**Table S3.** Commercial processed products assayed in the study.

| **No.** | **Form** | **Batch No.** | **Place of Origin** | **Manufacturer** |
| --- | --- | --- | --- | --- |
| 1 | Powder | 160302 | Xinjiang Province, PRC | Hua Dong Co., Ltd., PRC |
| 2 | Powder | 170322 | Xinjiang Province, PRC | Li Tong Feng Co., Ltd., PRC |
| 3 | Powder | 2016120026 | Xinjiang Province, PRC | Shang Hai TCM Co., Ltd.,PRC |
| 4 | Powder | 20141219 | Xinjiang Province, PRC | Xin Shi Ji Co., Ltd, PRC |
| 5 | Slice | 20170409 | Russia | ZTP Ginseng Birdnest Pte Ltd., Singapore |
| 6 | Powder | 091120 | Xinjiang Province, PRC | Zhong Tian Co., Ltd. |
| 7 | Powder | 20170410 | Unknow | Bo Zhou TCM shop |
| 8 | Powder | 20170412 | Unknow | Yu Xiang Tang TCM shop, PRC |
| 9 | Powder | 20180325 | Unknow | Yu He Tang TCM shop |
| 10 | Slice | 20180806 | Unknow | Jiu Lin TCM shop |
| 11 | Powder | 20180915 | Unknow | Yu Cao Tang TCM shop,PRC |
| 12 | Powder | 180102 | Inner Mongolia Province, PRC | Kang Mei Co., Ltd.,PRC |

**Table S4.** Results of species identification in commercial processed antelope horn products. The results of the multiplex PCR assay were reported as strongly positive (++), weekly positive (+) or negative (-).

| **No.** | **PST** | **POA** | **PCH** | **PPG** |
| --- | --- | --- | --- | --- |
| **1** | **++** | **－** | **－** | **－** |
| **2** | **++** | **－** | **－** | **－** |
| **3** | **++** | **－** | **－** | **－** |
| **4** | **++** | **－** | **－** | **－** |
| **5** | **++** | **－** | **－** | **－** |
| **6** | **++** | **++** | **－** | **－** |
| **7** | **－** | **++** | **++** | **－** |
| **8** | **－** | **++** | **+** | **－** |
| **9** | **++** | **++** | **+** | **－** |
| **10** | **－** | **++** | **－** | **－** |
| **11** | **++** | **－** | **－** | **－** |
| **12** | **++** | **－** | **－** | **－** |
